# Effect of smoking on serum xanthine oxidase, malondialdehyde, ascorbic acid and α-tocopherol levels in healthy male subjects

**DOI:** 10.12669/pjms.311.6148

**Published:** 2015

**Authors:** Ali Akbar Shah, Fatehuddin Khand, Tayyab Uddin Khand

**Affiliations:** 1 Ali Akbar Shah, M.Phil. Assistant Professor, Department of Biochemistry, Isra University Hyderabad, Sindh, Pakistan.; 2 Fatehuddin Khand, PhD. Professor, Department of Biochemistry, Isra University Hyderabad, Sindh, Pakistan.; 3 Tayyab Uddin Khand, M.Phil. Assistant Professor, Department of Biochemistry, Indus Medical College, Tando Mohammad Khan, Sindh, Pakistan.

**Keywords:** Ascorbic acid, Alpha-Tocopherol, Malondialdehyde, Smokers, Xanthine oxidase

## Abstract

**Objective:**

To examine the effect of smoking on serum xanthine oxidase, malondialdehyde, α- tocopherol and ascorbic acid levels in healthy adult male subjects.

**Methods:**

This cross-sectional comparative study was carried out at Isra University Hyderabad from July 2012 to December 2012. One hundred and twenty apparently healthy adult male subjects (60 smokers and 60 non-smokers) included in present study, were recruited from Jaindal kot, a small village located midway between Hyderabad and Matiari. Serum samples from smokers and non-smokers were analyzed for xanthine oxidase and malondialdehyde levels by standard kit methods, while for ascorbic acid and alpha- tocopherol by spectrophotometric methods.

**Results:**

The mean xanthine oxidase and malondialdehyde levels measured in healthy smokers were 0.30±0.05 mg/dl and 37.50±4.05 µmoles/l respectively as against 0.25±0.04 mg/dl and 19.86±2.21µmoles/l in non-smokers. Both xanthine oxidase and malondialdehyde levels were significantly (p<0.001) raised in healthy smokers than in non-smokers. Likewise, mean vitamin E and vitamin C levels were respectively 0.69±0.37 mg/dl and 0.80±0.16 mg/dl in healthy smokers compared to 1.14±0.43 mg/dl and 1.22±0.29 mg/dl in non-smokers. The concentrations of both these vitamins were significantly (p<0.001) lower in smokers than in non-smokers.

**Conclusion:**

The results of present study demonstrate that smoking had significantly increased xanthine oxidase and malondialdehyde levels and decreased vitamins C and E (antioxidants) levels. These findings suggest that smokers have to take additional amounts of vitamins C and E in order to avoid deleterious effects of smoking on their health.

## INTRODUCTION

The harmful effects of cigarette smoking on human health have been well documented.^1^^,^^2^ It has been known that cigarette smoke carries around 4000 chemicals including toxic metals, poisonous gases and free radicals.^3^ Amongst these constituents, free radicals are considered to be more dangerous as these owing to their unpaired electron are highly reactive and can cause oxidative damage to biomolecules and biomembranes.^4^ In addition to cigarette smoke, the other main external sources for free radicals include air pollutants and industrial wastes.^5^^,^^6^ In human body free radicals are normally produced as a result of regular metabolic processes such as mitochondrial respiration, arachidonic pathway, cyclooxygenase and lipoxygenase pathways.^7^ Generally free radicals generated inside the body are neutralized by antioxidants- molecules that give up their own electrons to free radicals to make them inactive and hence unable to cause damage to biological membranes by lipid peroxidation.^8^

Vitamin E, a well-known chain-breaking antioxidant in cell membrane protects the membrane against lipid peroxidation either directly by scavenging the free radicals or indirectly by controlling the reduced glutathione levels.^9^^-^^11^ Vitamin E itself is kept in reduced state by vitamin C, another antioxidant vitamin.^12^

It is now well recognized that when production of free radicals exceeds the antioxidant capacity in human body, then these can produce oxidative stress which is mainly responsible for the pathogenesis of several diseases such as cardiovascular, Alzheimer’s, Parkinson’s, and cancers.^13^^-^^17^

The purpose of present study was to examine the effect of cigarette smoke (a source of free radicals and toxic metals) on human health by comparing xanthine oxidase (a source of reactive oxygen species (ROS)), malondialdehyde (a marker for oxidative stress) and vitamins E and C (antioxidants) levels in blood samples of smokers and non-smokers.

## METHODS

One hundred and twenty apparently healthy male subjects, of whom 60 were smokers (age range: 24-39 years) and 60 non- smokers (age range: 21-39 years) were recruited from Jaindal Kot, a small village located midway between Hyderabad and Matiari. Smokers were those who had smoking history of over 10 cigarettes per day, while non-smokers were those who have not been smoking first hand.

Subjects who were either below 20 or over 40 years age; who were with a history of an ongoing chronic disease or those who were taking vitamin supplements prior to the study were excluded from this study.

Serum samples of smokers and non-smokers were analyzed for xanthine oxidase (XO) and malondialdehyde (MDA) levels by Eliza kits, while for vitamins C and E by spectrophotometric methods.^18^^,^^19^ Informed written consent was obtained from every subject involved in the study and the ethical approval of the study was granted by the Ethical Committee of Isra University.

***Statistical Analysis: ***Serum XO, MDA, vitamin C and vitamin E concentrations are presented as Mean ± SD and student’s t test (2 tailed) is used to compare the means between smokers and non-smokers. All the data was calculated on 95% confidence interval. P-value ≤0.05 was considered as statistically significant.

## RESULTS

In Table-I mean serum XO and MDA levels are compared between healthy adult male smokers and non-smokers, while in Table-II mean serum vitamin E and vitamin C levels are compared between the subjects of two groups.

Xanthine oxidase levels were found to be significantly (p<0.001) higher in smokers (0.30 ± 0.05 mg/dl) as against the non-smokers (0.25 ± 0.04mg/dl). Similarly, MDA levels were seen to be significantly (p<0.001) higher in smokers (37.50 ± 4.05 µmoles/l) than in non-smokers (19.86 ± 2.21 µmoles/l). 

Vitamin E levels were significantly (p<0.001) lower in smokers (0.69 ± 0.37 mg/dl) compared to non-smokers (1.14 ± 0.43 mg/dl). Similarly, Vitamin C levels were significantly (p<0.001) lower in smokers (0.80 ± 0.16 mg/dl) than in non-smokers (1.22 ± 0.29 mg/dl). The results of present study demonstrate that smoking had significantly increased XO and MDA levels and decreased vitamins C and E (antioxidants) levels.

## DISCUSSION

In this study serum XO and MDA levels were found to be significantly (p<0.001) raised in healthy adult male smokers than in non-smokers. High serum levels of XO in smokers could have amplified production of reactive oxygen species (ROS) which in turn might have accelerated lipid peroxidation manifested by increased levels of MDA in smokers compared to non-smokers.^20^ Smoking in this way could have caused tremendous damage to the cells through increased cell turnover leading to increased purine catabolism and hence increased ROS production.^16^^,^^21^

The role of smoking and alcohol intake on the activity of XO in patients with acute myocardial infarction (AMI) were studied by Kamble et al.^22^, who found that smokers were most likely to develop AMI than alchoholics.

In present study serum levels of both vitamins E and C were significantly (p<0.001) decreased in smokers compared to non-smokers. Our results about vitamins E and C are consistent with the reports of Duthie et al.^11^; Kelly^12^ and Kashinakunti et al.^20^ respectively. These findings suggest that smokers have to take additional amounts of both these vitamins in order to counteract smoking induced ROS production, and hence prevent harmful effects of smoking on their health.

Our results regarding the effect of cigarette smoke on the levels of XO, MDA and vitamins C and E clearly demonstrate that smoking increases oxidative stress by increasing the level of xanthine oxidase and decreasing the concentrations of antioxidant vitamins C and E.^17^ In this way, smokers encounter a sustained free radical load, which could enhance LDL oxidation and hence facilitate the development of the atheromatous plaque.^23^

**Table-I:** Comparison of serum XO and MDA levels between smokers and non-smokers

**Variable**	**Smokers** **N = 60**	**Non –smokers** **N= 60**	**Level of significance**
Xanthine oxidase (mg/dl)	0.30±0.05	0.25±0.04	P<0.001
Malondialdehyde (µmole/l)	37.50±4.05	19.86±2.21	P<0.001

N= Total number of subjects,

Data are presented as the mean±S.D

**Table-II:** Comparison of serum vitamins E and C levels between smokers and non – smokers

**Variable**	**Smokers** **N = 60**	**Non –Smokers** **N = 60**	**Level of significance**
Vitamin E (mg/dl)	0.60±0.37	1.14±0.43	P<0.001
Vitamin C (mg/dl)	0.80±0.16	1.22±0.29	P<0.001

N= Total number of subjects,

Data are presented as the mean ± S.D

## CONCLUSION

The results of present study demonstrate that smoking significantly increases **XO** and MDA levels and decreases vitamins C and E (antioxidants) levels. These findings suggest that smokers need to take additional amounts of these vitamins in order to avoid deleterious effects of smoking on their health.

## Authors Contribution:

**AAS: **Performed laboratory analyses and helped in manuscript writing.

**FDK: **Conceived and designed the study, made final critical revision of the manuscript for important intellectual content.

**TUK: **Did statistical analysis and editing of manuscript.
